# Methotrexate Therapy for Ectopic Pregnancies: A Tertiary Center Experience

**DOI:** 10.1055/s-0038-1675807

**Published:** 2018-11

**Authors:** Ozgur Ozyuncu, Atakan Tanacan, Sinem Ayse Duru, Mehmet Sinan Beksac

**Affiliations:** 1 Division of Perinatology, Department of Obstetrics and Gynecology, Faculty of Medicine at Hacettepe University, Ankara, Turkey

**Keywords:** methotrexate, ectopic pregnancy, medical gynecology, diagnosis

## Abstract

**Objective** Our aim is to demonstrate the importance of methotrexate (MTX) therapy for the treatment of ectopic pregnancy (EP).

**Methods** This retrospective study consisted of 99 patients (72 tubal EPs, 20 pregnancies of unknown location (PUL), 4 cesarean section (CS) scar EPs and 3 cervical EPs) treated with MTX.

**Results** Methotrexate therapy was successful in 68.5% of EPs. There were statistically significant differences between the MTX success and failure groups based on ultrasonographic findings, patient complaints, gestational week and serum human chorionic gonadotropin (hCG) values. The MTX success rates in PUL and tubal pregnancies were 95% and 61.1%, respectively. The MTX success rates in single-dose, two-dose and multi-dose protocol groups were 86.9%, 28.6% and 40%, respectively. All cervical and CS scar ectopic pregnancies were treated successfully with MTX therapy.

**Conclusion** Methotrexate might be the first-line treatment option for EPs under certain conditions. Physicians must be more cautious in cases with higher hCG values, the presence of abdominal-pelvic pain, the presence of fetal cardiac activity, larger gestational sac (GS) diameters, and more advanced gestational weeks according to the last menstrual period.

## Introduction

Ectopic pregnancy (EP) is defined as the implantation of the blastocyst outside the uterine cavity.^1^ It is the foremost cause of maternal mortality, which accounts for 4.9% of maternal deaths in developed countries.^2^ The rate of EP is ∼ 1 to 2%.^3^ Approximately 98% of EPs occur in the fallopian tube but can also be located in the cervix, cornual side of the fallopian tubes (interstitial), cesarean scar, intramural portion of the fallopian tubes, ovaries, or abdomen. Furthermore, EP can be heterotopic, that is, both intrauterine and extrauterine pregnancies together.^4^

The most common clinical signs and symptoms of EP are vaginal bleeding and abdominal-pelvic pain, although EP can also be asymptomatic.^5^ Vaginal bleeding, pelvic and/or abdominal pain together with improper serial human chorionic gonadotropin (hCG) values (no doubling, low reduction, or plateaued levels at 48 hours) are indicative for EP. Additionally, a transvaginal ultrasound (ultrasonographic findings such as extrauterine gestational sac and/or hematosalpinx with empty endometrial cavity) may be used as an ancillary imaging method for the diagnosis of EP. However, the diagnosis of EP is mainly clinical and not based on ultrasound findings.^6^ ^7^ Serum progesterone concentrations, serum creatine kinase concentrations, specimens obtained from curettage procedures, and diagnostic laparoscopy operations are other ancillary interventions that can be used for diagnosis.^8^ ^9^ ^10^ ^11^ Pregnancy of unknown location (PUL) is the preferred terminology when the serum hCG is positive, but no signs of intrauterine or extrauterine pregnancy can be detected using transvaginal ultrasonography.^12^ Appropriate management of EP is crucial to prevent patient mortality and morbidity related to EP rupture.^2^ ^13^

Methotrexate (MTX), which is a folinic acid antagonist, has been used as a first-step therapy in the treatment of EP for years.^7^ ^14^ Systemic MTX can be given in single-, double-, and multi-dose protocols depending on the clinical findings and beta-human chorionic gonadotropin (β-hCG) values.^7^ ^11^ In special circumstances, like interstitial, cervical, cesarean scar, and ovarian EPs, an intra-amniotic MTX may also be administered. 15 The success rate of MTX is ∼ 90% in properly selected patients and leads to fewer complications, adverse treatment outcomes, and lower economic cost when compared with surgical interventions.^16^ ^17^

In this study, our aim was to share our experience with MTX therapy in a tertiary care center and to evaluate the factors that affect MTX therapy success rates.

## Methods

We retrospectively evaluated the data of 99 patients to whom MTX was given for the treatment of EP at the Department of Obstetrics and Gynecology of the Hacettepe University Hospital, between January 2008 and September 2017. The required data were extracted from the Hacettepe University patient database. Ectopic pregnancy was diagnosed by experienced physicians based on transvaginal ultrasonographic findings (extrauterine gestational sac and/or hematosalpinx with empty endometrial cavity), clinical signs (vaginal bleeding, and pelvic and/or abdominal pain), and improper serial hCG values (no doubling, low reduction, or plateaued levels at 48 hours). Patients with EP who were suitable for MTX treatment were included in the study. The eligibility criteria for MTX therapy were as follows: 1) hemodynamically stable patients, 2) no contraindications to MTX therapy (absence of hematologic, renal, hepatic, pulmonary diseases, active peptic ulcer, hypersensitivity to MTX, or currently breastfeeding), 3) hCG concentration ≤5,000 mIU/ml (for interstitial, cervical, and cesarean scar EPs, this criterion was not accepted), 4) patients must have been able to coordinate with physicians for measurement of serial hCG levels following treatment and access emergency services in case of EP rupture with signs and symptoms of acute abdomen and hemodynamic instability. Patients who did not fulfill these conditions were excluded from the study.

Either a single-dose protocol of 1-mg/kg intramuscular MTX, two-dose protocol of 1-mg/kg intramuscular MTX on days 0 and 4 or a multi-dose protocol of 1-mg/kg MTX per day on days 1, 3, 5, and 7 with folinic acid 0.1 mg/kg on days 2, 4, 6, and 8 were instituted depending on the EP characteristics and physician's experience. In special circumstances, such as ectopic scar pregnancy and cervical ectopic pregnancy, intra-amniotic MTX therapy (25 mg) was also administered in combination with a 20% solution of potassium chloride (1–5 ml), in addition to the systemic MTX therapy.

The success of treatment protocols was evaluated depending on the percentage of decline in hCG values between days 4 and 7, which must be > 15%. If there was a < 15% decline, an additional dose was applied in addition to the preferred protocol. Medical treatment failure was defined as the necessity for surgical interventions. If hCG levels did not decline properly, plateaued, or increased despite the additional MTX doses, and/or in the presence of EP rupture with signs and symptoms of acute abdomen and hemodynamic instability, surgical interventions were performed. The levels of hCG were checked weekly until the level was undetectable in venous blood samplings to confirm complete medical treatment success.

To provide consistent results, we evaluated ectopic scar pregnancies and cervical pregnancies separately, which needed a different treatment protocol. Cases in this category consisted of three cervical and four cesarean scar EPs. The remaining patients with tubal or PUL EPs (92 patients), were divided into two groups according to medical therapy success or failure. The demographic and clinical characteristics of the patients (age, gravida, parity, previous abortions, history of previous EP, EP localization depending on the ultrasonographic finding, hCG levels, percentage of hCG level decline between days 4 and 7, gestational sac [GS] diameter, patient complaint, gestational week according to the last menstrual period, MTX dosage, MTX therapy success/failure, and surgical interventions) were compared between the groups. Furthermore, the MTX success rates of PUL and tubal pregnancies were calculated. Finally, the therapy success rates of single-dose, two-dose and multi-dose MTX protocols were determined. Ectopic pregnancy cases were treated with the single-dose protocol in 61 patients (66.3%), two dose-protocol was used in 21 patients (22.8%), and the multidose protocol was used in 10 patients (10.9%).

Statistical analyses were performed with the software SPSS Statistics for Windows, Version 22.0. (IBM Corp., Armonk, NY, USA). The Kolmogorov-Smirnov test was used to evaluate the normal distribution of the data. Normally distributed data were presented as means and standard deviations, while non-parametric data were presented as median (range) values. The independent-samples t-test and Mann-Whitney U-test were used to compare the parametric and non-parametric variables between the groups, respectively; the categorical variables were compared using the chi-squared test. The significance level with a *p* value of < 0.05 was determined. Written informed consent was obtained from all the patients, and the study was approved by the institutional ethics committee of Hacettepe University. No funding was used for this study.

## Results

Seventy-two patients had tubal EPs (72.7%), 20 patients had PUL (20.2%), 3 patients had cervical EPs (3%), and 4 patients had cesarean scar EPs (4.1%). Cervical and cesarean scar ectopic pregnancies were treated with multi-dose protocol MTX therapy together with 25 mg intra-amniotic MTX and a 20% solution of potassium chloride (1–5 ml). The remaining EP cases were treated with the single-dose protocol in 61 patients (66.3%), two doses of MTX were needed in 21 patients (22.8%), and the multi-dose protocol was used in 10 patients (10.9%).

To provide consistent results, we evaluated ectopic scar and cervical pregnancies separately. The mean and standard deviation values for numerical variables together with percentages for categorical variables of patient characteristics, obstetric history variables, and clinical findings are shown in Table 1. All of these EPs (three cervical and four cesarean scar EPs) were successfully treated with medical therapy, and no further surgical interventions were needed.

**Table 1:** The mean and standard deviation values for numerical variables together with percentages for categorical variables of patient characteristics, obstetric history, and clinical findings in cervical and cesarean scar ectopic pregnancy groups

Variables		Cervical EP (*n* = 3)	Cesarean scar EP (*n* = 4)
Maternal age (years)		31.00 ± 2.65	31.75 ± 6.70
Gravida		2.67 ± 0.58	2.25 ± 0.96
Parity		1.33 ± 0.58	1.25 ± 0.96
Abortus		0.33 ± 0.58	0.00 ± 0.00
History of previous EP		None	None
Patient's complaint	Vaginal bleeding	3/3 (100%)	3/4 (75%)
	Abdominal-Pelvic pain	0/3 (0%)	1/4 (25%)
GS diameter (mm)		30.00 ± 13.23	19.00 ± 8.54
Fetal cardiac activity		3/3 (100%)	3/4 (75%)
Gestational week according to the last menstrual period		8 ± 1.00	7.25 ± 0.96
hCG value on the first day (mIU/ml)		46,711.33 ± 40,474.17	49,654.35 ± 41,154.22
hCG value on the fourth day (mIU/ml)		34,783.17 ± 26,851.06	33,650.21 ± 27,854.23
hCG value on the seventh day (mIU/ml)		27,014.75 ± 20,850.24	26,561.23 ± 21,577.19

Abbreviations: EP, ectopic pregnancy; GS, gestational sac; hCG, human chorionic gonadotrophin.

The remaining EP patients (72 tubal and 20 PUL) were divided into two groups determined by the success or failure of the MTX treatment. Sixty-three EPs were successfully treated with MTX treatment (68.5%), while surgical procedures were necessary in 29 patients (31.5%). Table 2 shows the median and range values for numerical variables together with percentages for categorical variables of patient characteristics, obstetric history variables, and clinical findings compared between the MTX treatment success and failure groups.

**Table 2:** The median and range values for numerical variables together with percentages for categorical variables of patient characteristics, obstetric history variables, and clinical findings compared between the MTX treatment success and failure groups

Variables		MTX success group (*n* = 63)	MTX failure group (*n* = 29)	*p*-values
USG findings	Tubal	44/63 (69.8%)	28/29 (96.5%)	0.08
	PUL	19/63 (30.2%)	1/29 (3.5%)	0.001
Maternal age (years)		31 (24–42)	31 (26–43)	0.98
Gravida		3 (1–5)	3 (1–5)	0.62
Parity		1 (0–2)	1 (0–2)	0.50
Abortus		1 (0–2)	0 (0–1)	0.37
History of previous EP		8/63 (12.7%)	9/20 (31%)	0.06
Patient's complaint	Vaginal bleeding	52/63 (82.5%)	14/29 (48.3%)	0.001
	Abdominal-pelvic pain	11/63 (17.5%)	15/29 (51.7%)	0.001
GS diameter (mm)		8 (3–15)	13 (5–20)	0.001
Fetal cardiac activity		1/63 (1.5%)	7/29 (24.1%)	< 0.001
Gestational week according to the last menstrual period		5 (4–8)	7 (4–8)	< 0.001
hCG value on the first day (mIU/ml)		2,400 (100–4,800)	4,200 (216–4,950)	< 0.001
hCG value on the fourth day (mIU/ml)		2,000 (75–4,700)	4,100 (210–4,900)	< 0.001
hCG value on the seventh day (mIU/ml)		841 (12–3,000)	3,850 (340–5,100)	< 0.001
Percentage for median of hCG decline between days 4 and 7		51.12%	7.14%	< 0.001

Abbreviations: EP, ectopic pregnancy; GS, gestational sac; hCG, human chorionic gonadotrophin; MTX, methotrexate; PUL, pregnancy of unknown location; n, number of patients, USG, ultrasonography.

There were no statistically significant differences between MTX success and failure groups for the medians of maternal age, gravida, parity, abortus, and percentage of history of previous EP (*p* = 0.98, *p* = 0.62, *p* = 0.50, *p* = 0.37, and *p* = 0.06, respectively). There were statistically significant differences between MTX success and failure groups for the percentage of ultrasonographic findings compatible with PUL; patient complaint (vaginal bleeding versus abdominal-pelvic pain); fetal cardiac activity; median GS diameter; gestational week according to the last menstrual period; hCG values on the first, fourth, and seventh days of treatment; and the percentage of decline in hCG level between days 4 and 7 (*p* = 0.001, *p* = 0.001, *p* = 0.001, *p* < 0.001, *p* = 0.001, *p* < 0.001, *p* < 0.001, *p* < 0.001, *p* < 0.001, and *p* < 0.001, respectively).

Pregnancy of unknown location was diagnosed in 30.2% of patients in the MTX success group, while the percentage was only 3.5% in the MTX failure group. Vaginal bleeding was documented in 82.5% of the MTX success group and was documented in 48.3% of the MTX failure group. Furthermore, abdominal-pelvic pain was reported in 51.7% of the MTX failure group, but only reported in 17.5% of the MTX success group. Fetal cardiac activity was observed in 24.1% of the MTX failure group patients but was observed in only 1 patient (1.5%) in the MTX success group. The median GS diameter, gestational week according to last menstrual period, and hCG values on days 1, 4, and 7 of the MTX treatment were all higher, and the percentage of hCG level decline between days 4 and 7 was significantly lower in the MTX failure group than in the MTX success group.

In this study, the MTX success rates in PUL and tubal pregnancies were 95% (19/20) and 61.1% (44/72), respectively. The MTX success rates in single-dose, two-dose and multi-dose protocol groups were 86.9% (53/61), 28.6% (6/21) and 40% (4/10), respectively (Table 3).

**Table 3:** Methotrexate success rates in single-dose, two-dose and multi-dose protocol groups

MTX Group (n)	Success rate
Single-dose (61)	86.9% (53/61)
Two-dose (21)	28.6% (6/21)
Multi-dose (10)	40% (4/10)

Abbreviations: MTX, methotrexate; n, number of patients.

## Discussion

Expectant management, medical treatment, and surgical approaches are the options for the management of EP, as advances in early diagnosis have been achieved in recent decades.^18^ In hemodynamically stable patients, MTX is a safer and less expensive alternative to surgery.^16^ ^17^ The single-dose protocol was the first-line therapy for EP in selected cases for many years.^18^ ^19^ The multi-dose protocol has more adverse effects and has similar success rates when compared with single-dose protocols in recent studies.^20^ ^21^ However, the double-dose protocol has been introduced to combine the effectiveness and safety of single- and multi-dose protocols.^22^ In our series, we have demonstrated that success rates were 86.9% (53/61), 28.6% (6/21) and 40% (4/10), respectively, for single-dose, two-dose and multi-dose MTX protocols, respectively.

Methotrexate is a folinic-acid antagonist that inactivates DNA synthesis, especially in fast-growing tissues.^23^ Definitive contraindications for MTX therapy were hemodynamic instability, signs and symptoms of acute abdomen with EP rupture, heterotopic pregnancy, breastfeeding, MTX sensitivity, active peptic ulcer disease, presence of immunodeficiency, hepatic, renal, and hematologic diseases, and in some occasions, higher levels of hCG (with > 5,000 mIU/ml as a relative contraindication).^5^ ^7^ ^14^ ^17^ ^18^ Adverse effects such as nausea, vomiting, stomatitis, elevated liver function tests, anorexia, and diarrhea may be observed during the treatment.^23^ The success of medical treatment is considered to be achieved when a decline >15% in the hCG level between days 4 and 7 is obtained, and complete hCG resolution occurs during the follow-up process.^7^ ^24^

Initial hCG concentrations, localization of EP, clinical signs/symptoms, and ultrasonographic findings are the main factors that affect the success rate of the MTX treatment.^25^ In our study, there were statistically significant differences between the MTX success and failure groups for the percentage of ultrasonography findings compatible with PUL; patient complaint (vaginal bleeding versus abdominal-pelvic pain); fetal cardiac activity and median GS diameter; gestational week according to the last menstrual period; hCG values on the first, fourth, and seventh days of treatment; and percentage of decline in hCG level between days 4 and 7 (*p* = 0.001, *p* = 0.001, *p* = 0.001, *p* < 0.001, *p* = 0.001, *p* < 0.001, *p* < 0.001, *p* < 0.001, *p* < 0.001, and *p* < 0.001, respectively). Pregnancy of unknown location was diagnosed in 30.2% of the MTX success group, while the ratio was only 3.5% in the MTX failure group. Vaginal bleeding was reported in 82.5% of the patients in the MTX success group and 48.3% of the patients in the MTX failure group. Furthermore, abdominal-pelvic pain was reported in 51.7% of the patients in the MTX failure group and in 17.5% of the patients in the MTX success group. Fetal cardiac activity was observed in 24.1% of the patients in the MTX failure group but was observed in only 1 patient (1.5%) in the MTX success group. The median GS diameter, gestational week according to last menstrual period, and hCG values on days 1, 4, and 7 of MTX treatment were all higher in the MTX failure group than in the MTX success group. These findings were compatible with the current literature.^26^ ^27^

Three cervical and four cesarean scar EPs were successfully treated with medical therapy and no further surgical interventions were needed in our study. This is an important point because cervical and cesarean scar EPs are complicated cases that can cause abundant bleeding, with life-threatening conditions that require surgery in most situations.^28^ ^29^ Intra-amniotic MTX and 20% potassium chloride solution injection together with a systemic multi-dose protocol can be a beneficial alternative to surgery.

## Conclusion

In conclusion, MTX might be the first-line treatment option in EPs under proper conditions (in hemodynamically stable patients without a contraindication to MTX therapy). Physicians must be more cautious in cases with higher hCG values, abdominal-pelvic pain signs and symptoms, the presence of fetal cardiac activity, larger GS diameters, and higher gestational weeks according to the last menstrual period, as medical therapy has a higher risk of failure and surgery may be needed in these cases.
